# A Case of an Acute Zonal Occult Outer Retinopathy Variant Characterized With an Insidious Peripheral Onset and Centripetal Progression

**DOI:** 10.7759/cureus.59600

**Published:** 2024-05-03

**Authors:** Barbaros Hayrettin Ünlü, Omer Karti, Ali O Saatci

**Affiliations:** 1 Department of Ophthalmology, Dokuz Eylül University, Izmir, TUR

**Keywords:** otoimmune disorders, optical coherence tomography, outer retinal loss, fundus autofluorescence, acute zonal occult outer retinopathy

## Abstract

Acute zonal occult outer retinopathy (AZOOR) manifests as the rapid loss of one or multiple large zones of the outer retinal layers, often with a distinct sectoral distribution. Subtle fundus changes, such as pigmentary alterations around the optic nerve, are typically present in the early stages. Disease progression is characterized by the appearance of well-defined atrophic zones involving the outer retina, retinal pigment epithelium, and choroid. AZOOR lesions typically begin in the peripapillary region and then spread centrifugally toward the peripheral fundus. In this case report, we present the clinical and multimodal imaging characteristics of a 63-year-old woman with a symmetrical, peripheral-onset AZOOR variant with a very slow centrifugal progression. Most notably, the posterior pole was unaffected bilaterally.

## Introduction

The term acute zonal occult outer retinopathy (AZOOR) describes the rapid loss of one or multiple large zones of the outer retinal layers, often presenting with a distinctive sectoral distribution [1]. During the initial stages, patients usually exhibit minimal noticeable fundus changes, such as subtle pigmentary alterations in the peripapillary zone, alongside visual symptoms, such as photopsia, decreased contrast sensitivity, and photophobia. As the disease progresses, the lesion becomes more evident, with a well-defined and sequentially demarcated atrophic zone affecting the outer retina, retinal pigment epithelium (RPE), and choroid [1-4].

AZOOR lesions commonly originate in the peripapillary zone and tend to progress centrifugally toward the peripheral fundus [1-4]. This case report aims to delineate the clinical and multimodal imaging characteristics of a 63-year-old female with a symmetric, peripheral-onset AZOOR variant demonstrating a very slow centrifugal progression. Most notably, the posterior pole was unaffected bilaterally.

## Case presentation

**Figure 1:**
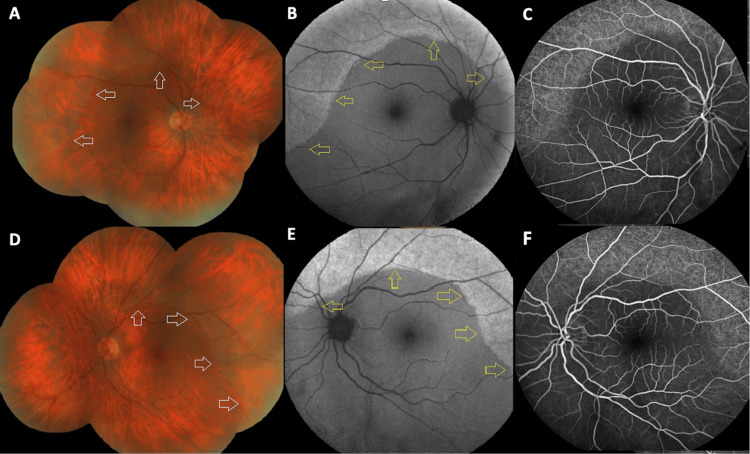
The color fundus, fundus autofluorescence, and fluorescein angiographic images of the right and left eyes. Color fundus pictures of the right (A) and left (D) eyes showing subtle, symmetric, grayish-white retinal lesions located circumferentially at the periphery. The affected retinal area was distinctly demarcated from the intact retina by a continuous yellow-orange curvilinear line marked with white arrows. Fundus autofluorescence images of the right (B) and left (E) eyes displaying diffuse hyperautofluorescent lesions at the affected peripheral retina, indicated by yellow arrows. Fluorescein angiographic images of the right (C) and left (F) eyes depicting a hyperfluorescent appearance at the affected peripheral retina.

**Figure 2:**
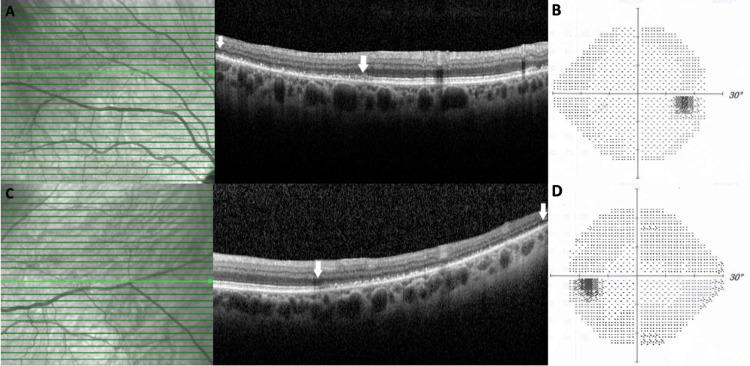
Spectral domain optical coherence tomography (SD-OCT) scan and visual field test of the right and left eyes. SD-OCT sections through the affected retina, demarcation line, and unaffected retina of the both right (A) and left (C) eyes revealing the loss of the outer retina within the affected retina, as indicated by the region between the white arrows. Visual field of the right eye (B) and left eye (D) demonstrating the peripheral visual field depression.

**Figure 3:**
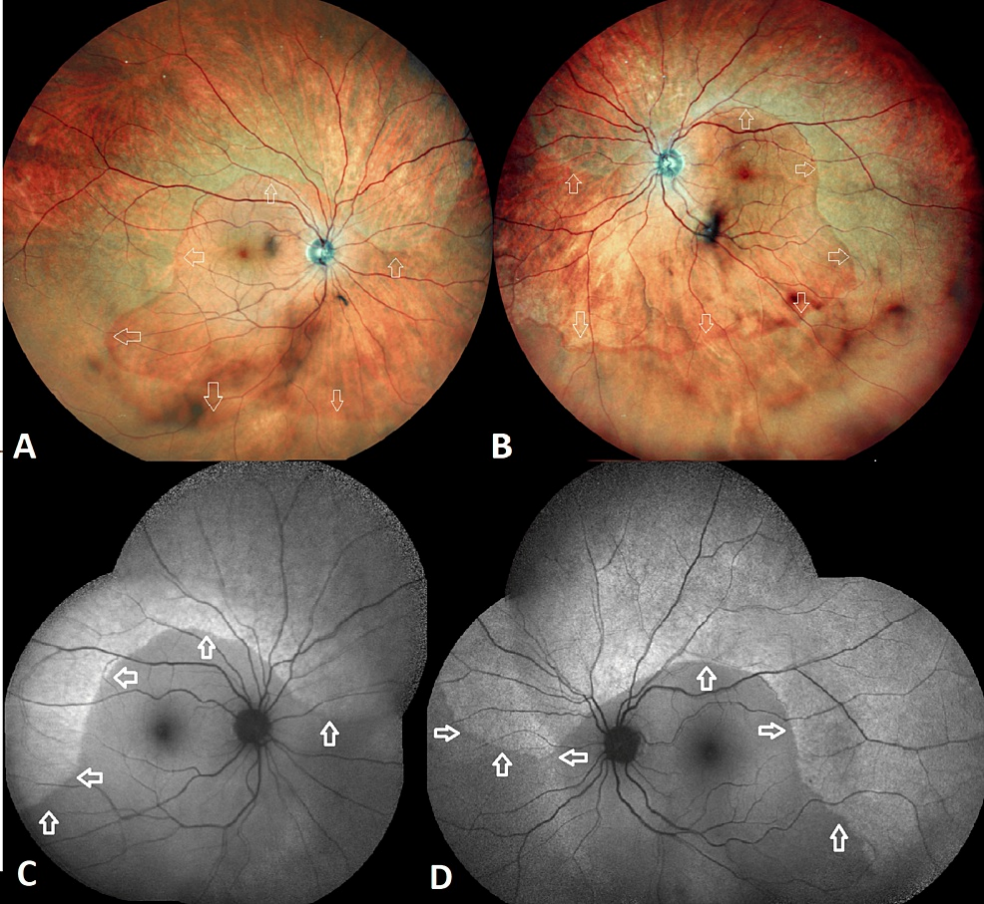
Wide-field color fundus and fundus autofluorescence images of the right and left eyes at the first-year follow-up. Wide-field color fundus pictures (NIDEK Mirante; Nidek Co., Ltd., Gamagori, Japan) of the right (A) and left (B) eyes revealing a very mild centripetal progression of the peripheral lesions toward the center with an intact posterior pole a year later. The demarcation line, separating the affected and unaffected retinal areas, is indicated by the white arrows. Fundus autofluorescence images of the right (C) and left (D) eyes demonstrating a very mild centripetal progression of the diffuse hyperautofluorescent lesions, with the lesion borders marked by white arrows.

A 63-year-old visually asymptomatic woman was referred to us for a routine screening visit to check for possible hydroxychloroquine toxicity. She had previously received a diagnosis of systemic lupus erythematosus and was on oral leflunomide (for two years) and hydroxychloroquine (for nine years). She also had a medical history of asthma and hypothyroidism. The best-corrected Snellen visual acuity was 10/10 (-2.0 diopters) in OD and 9/10 (-1.50 diopters) in OS. Slit-lamp examination was unremarkable OU, and intraocular pressure was within normal limits bilaterally. Fundoscopy revealed circumferential grayish-white looking symmetric peripheral fundus lesions OU. These lesions did not involve the posterior pole. The border between the affected peripheral retinal area and the unaffected posterior pole was visible as a continuous curvilinear yellow-orange line (Figures 1A, 1D). Fundus autofluorescence images revealed peripheric, circumferential, well-delineated, hyperautofluorescent areas in both eyes (Figures 1B, 1E). Fluorescein angiography showed hyperfluorescence at the corresponding peripheral fundus area (Figures 1C, 1F). Optical coherence tomography imaging revealed a loss of outer retinal structures, including the myoid, ellipsoid, and interdigitation zone. Although the external limiting membrane was intact outside of the affected zone, its integrity was compromised within the affected area. Additionally, both the outer nuclear and outer plexiform layers appeared thinner with a dot-like hyperreflective material accumulation within the photoreceptor layer (Figures 2A, 2C). Visual field testing indicated bilateral diffuse peripheral depression (Figures 2B, 2D). Blood biochemistry was normal and serological tests for syphilis, cytomegalovirus, herpes zoster, and herpes simplex yielded negative results. The diagnosis of a peripheral-onset AZOOR variant was reached. Hydroxychloroquine treatment was replaced with mycophenolate mofetil by her rheumatologist. A year later, the patient remained visually asymptomatic, and a minimal centripetal progression of the peripheral lesions was noted (Figures 3A-3D).

## Discussion

Typical AZOOR lesions are commonly localized at the peripapillary zone and tend to progress centrifugally toward the peripheral retina. However, concentric zonal involvement at the periphery has been rarely reported [5-7]. The current case represents an atypical variant of AZOOR where there was bilateral, symmetrical, circumferential zonal involvement at the peripheral fundus with a centripetal slow progression. Remarkably, both posterior poles remained intact during the one-year follow-up.

To our knowledge, there are only a few reported AZOOR cases featuring zonal peripheric retinal involvement besides peripapillary involvement. Mrejen et al. [5] reviewed the clinical characteristics of 48 eyes of 30 patients with AZOOR and 18 patients had bilateral involvement but only four of these 18 patients had symmetric bilateral lesions. Out of 48 eyes, 43 exhibited a demarcating line between the involved and uninvolved retina. In their study, every AZOOR patient had disease involvement in one or more zonal areas, with the peripapillary region being the most frequently affected area. However, only two patients exhibited peripheral involvement. One patient presented with a substantial peripapillary lesion alongside a smaller peripheral lesion, and another patient displayed two lesions in the mid-peripheral fundus.

Tan et al. [6]. reported three AZOOR patients with peripheral, concentric zonal involvement, showing a centripetal progression extending from the periphery to the posterior fundus. However, all six eyes of the three patients exhibited concurrent central peripapillary AZOOR lesions progressing in a centrifugal manner. In contrast, our case revealed no evidence of peripapillary lesions in both eyes.

Ramtohul et al. [7] described the clinical features of 20 eyes of 10 patients with multizonal outer retinopathy and retinal pigment epitheliopathy (an unusual variant of AZOOR). All eyes had bilateral both peripapillary and far peripheral retina lesions. Far peripheral lesions were characterized by well-demarcated, 360-degree annular zones of RPE atrophy accompanied by large spots of RPE hyperpigmentation in all eyes. Centrifugal and centripetal progression of the peripapillary and far peripheral lesions caused areas of complete outer retinal and retinal pigment epithelial atrophy during the disease course. The authors reported that an initial alteration of photoreceptors and RPE, along with a stereotypical natural course involving the far retinal periphery, characterizes this unusual condition. Consequently, they considered it might represent a variant of AZOOR or possibly a new entity. Thus, they proposed naming it multizonal outer retinopathy and retinal pigment epitheliopathy (MORR). However, our case displayed some deviations from MORR. While peripapillary involvement accompanied far peripheral involvement in all cases described in MORR, our case exhibited a symmetrical, peripheral onset with a slow centripetal progression, devoid of peripapillary involvement. Another notable difference was the absence of retinal pigment epitheliopathy or RPE alterations observed in MORR cases. Hence, as the multimodal imaging of our case did not resemble either MORR or the classical AZOOR picture, we conjectured that this case represented another end of the AZOOR spectrum.

## Conclusions

The present case exhibited unusual clinical features for the AZOOR spectrum. First, the patient was visually asymptomatic and only received the diagnosis upon a routine screening eye examination for the toxicity of hydroxychloroquine. Second, there was no concurrent peripapillary involvement. Third, bilateral symmetric lesions commenced at the periphery and demonstrated a very slow centripetal progression. In light of our case, clinicians should be aware of this very rare AZOOR variant that may reflect a slow centripetal progress.
